# Advances in genome sequencing and artificially induced mutation provides new avenues for cotton breeding

**DOI:** 10.3389/fpls.2024.1400201

**Published:** 2024-07-02

**Authors:** Peilin Wang, Mubashir Abbas, Jianhan He, Lili Zhou, Hongmei Cheng, Huiming Guo

**Affiliations:** ^1^ Nanfan Research Institute, Chinese Academy of Agricultural Sciences (CAAS), Sanya, Hainan, China; ^2^ Biotechnology Research Institute, Chinese Academy of Agricultural Sciences, Beijing, China; ^3^ Institute of Cereal and Oil Crops, Hebei Academy of Agriculture and Forestry Sciences, Hebei Key Laboratory of Crop Genetics and Breeding, Shijiazhuang, Hebei, China; ^4^ Yazhouwan National Laboratory, Sanya, Hainan, China

**Keywords:** cotton, sequencing, diversity, mutant library, germplasm resources

## Abstract

Cotton production faces challenges in fluctuating environmental conditions due to limited genetic variation in cultivated cotton species. To enhance the genetic diversity crucial for this primary fiber crop, it is essential to augment current germplasm resources. High-throughput sequencing has significantly impacted cotton functional genomics, enabling the creation of diverse mutant libraries and the identification of mutant functional genes and new germplasm resources. Artificial mutation, established through physical or chemical methods, stands as a highly efficient strategy to enrich cotton germplasm resources, yielding stable and high-quality raw materials. In this paper, we discuss the good foundation laid by high-throughput sequencing of cotton genome for mutant identification and functional genome, and focus on the construction methods of mutant libraries and diverse sequencing strategies based on mutants. In addition, the important functional genes identified by the cotton mutant library have greatly enriched the germplasm resources and promoted the development of functional genomes. Finally, an innovative strategy for constructing a cotton CRISPR mutant library was proposed, and the possibility of high-throughput screening of cotton mutants based on a UAV phenotyping platform was discussed. The aim of this review was to expand cotton germplasm resources, mine functional genes, and develop adaptable materials in a variety of complex environments.

## 1 Introduction

The landscape of genomics has undergone a profound transformation in recent years, thanks to the rapid progress in high-throughput genome sequencing technologies. These advancements have paved the way for a thorough examination of entire genomes, enabling the identification of genes and their associated biological functions (Ng and Kirkness, 2010). Consequently, the scientific community has been actively engaged in characterizing genes crucial for development, cellular processes, and responses to diverse stresses (Ng and Kirkness, 2010). Traditionally, the understanding of gene function relied on two distinct yet complementary approaches: forward genetics and reverse genetics. Forward genetics entails identifying phenotype variations arising from spontaneous or induced mutations. Subsequently, the responsible mutant genes and their functions are identified and characterized (Jill, 2016). Initially, this method leveraged naturally occurring mutants with specific traits to uncover and study the genes governing those traits (Schneeberger and Weigel, 2011). However, with the advent of cost-effective high-throughput genome sequencing, it has become more practical to intentionally induce mutations and explore their effects on gene function (Johannes and Schmitz, 2019).

To conduct comprehensive high-throughput functional research on plant genes, it is crucial to have an extensive collection of mutant materials covering a diverse range of plant species. Leveraging whole-genome sequence data and employing reverse genetics methods enable large-scale screening of mutant libraries, facilitating the systematic study and annotation of all genes within a genome (Hagen, 2000). The completion of whole-genome sequencing for the model plant *Arabidopsis thaliana* in 2000 (Arabidopsis Genome Initiative, 2000), coupled with the subsequent establishment of an Arabidopsis mutant library (Greene et al., 2003), marked a significant breakthrough that propelled genomics research into a new era. Similarly, the sequencing of the rice genome in 2002 (Yu et al., 2002) and the creation of the rice mutant library in 2006 (Li et al., 2006) ushered in unprecedented advancements in genomics research.

Cotton, serving as a primary source of renewable fiber globally, plays a crucial role in the textile manufacturing industry. The cotton genus encompasses 46 diploid (2n=2x=26) species and seven tetraploid (2n=4x=52) species. The diploid cotton species are thought to share a common ancestor, with genomes categorized into eight groups: A, B, C, D, E, F, G, and K. The tetraploid cotton species originated from hybridization between the A genome species *G. arboreum* (A_2_) and the D genome species *G. raimondii* (D_5_) (Huang et al., 2021). Significant strides have been made in cotton genomics in recent years. The draft genome of the diploid cotton *G. raimondii* (D_5_) (Wang et al., 2012), *G. arboreum* (A_2_) in 2014 (Li et al., 2014). *G. hirsutum* (AD), a tetraploid cotton species, was fully sequenced, along with the development of a high-density genetic map (Wang et al., 2015). These groundbreaking achievements in cotton genomics, including the sequencing of the complete cotton genome, have ushered in a new era in cotton functional genomics research.

The creation of cotton mutant libraries through diverse approaches is imperative given the notable progress in functional genomics. Diversifying cotton germplasm resources not only presents significant potential for generating stable, high-quality cotton materials, but also serves as the foundation for enhancing crop productivity, improving fiber quality, and developing varieties with increased resistance to biotic and abiotic stresses.

The main goals of this review are to thoroughly analyze the establishment of cotton mutant libraries and the sequencing methodologies utilized, as well as to emphasize the discovery of new germplasm resources and gene functions. Additionally, we suggest prospective directions for cotton mutant research and explore the methods used in creating cotton mutant libraries, encompassing the induction of mutations using ethyl methanesulfonate (EMS), a widely employed mutagen in plant genetics.

Our discussion covers an assessment of the advantages and limitations of EMS mutagenesis, along with an examination of the strategies used to screen and characterize mutants exhibiting altered traits. Moreover, we underscore the efficacy of next-generation sequencing technologies in scrutinizing the mutant population, pinpointing causative mutations, and unraveling the functional consequences at the genomic level.

## 2 Recent advances in cotton genome sequencing and EMS mutant library development

Cotton is a versatile natural textile fiber with great importance in the global economy. The genus Gossypium consists of 45 diploid (2n = 2x = 26) and 5 tetraploid (2n = 4x = 52) species. The tetraploid cotton species are believed to have originated from the interspecific hybridization between an A-genome diploid species (e.g. *G. herbaceum* or *G. arboreum*) and a D-genome diploid species (G. raimondii), eventually leading to the emergence of at least 5 allotetraploid AD genome species (Huang et al., 2021). Allopolyploid cotton, which includes the primary cotton species *G.hirsutum* and *G. barbadense*, is believed to have emerged in the last 1–2 million years through hybridization and subsequent polyploidization events between the progenitors of the A- and D-subgenomes (Wendel et al., 2009). The global cotton production is dominated by *G. hirsutum* due to its high yield potential, moderate fiber quality, and broad adaptability. In contrast, *G. barbadense*, renowned for its high-quality, long-staple cotton fiber, plays a vital role as a key raw material for high-grade textile production, despite its lower yield potential (Hu et al., 2019). However, the constrained environmental adaptability of *G. barbadense* poses a significant barrier to its widespread cultivation.

In nature, the continuous interplay of natural and artificial selection leads to the emergence of a pool of spontaneous mutations. While this pool contains a considerable number of mutations, the intricate genetic background of these mutants presents challenges for researchers. The complexity, coupled with the low frequency of spontaneous mutations, poses difficulties for high-throughput and large-scale functional genomics research. This challenge is particularly pronounced in tetraploid cotton, where natural mutations are scarce, significantly impeding the progress of functional genomics development.

The efforts to enhance functional genomics efficiency have been accelerated by advancements in genome sequencing, which over the past two decades, has led to the development of genetically modified cotton varieties with increased resistance to insects and herbicides (Guo et al., 2015; Yu et al., 2016). This progress is a testament to the collaborative efforts within the scientific community, as exemplified by the application of comprehensive whole-genome sequences from model organisms like Arabidopsis and rice. Such foundational work has significantly contributed to consortium-based cotton genome research, spearheaded by initiatives like the Cotton Genome Consortium strategic plan in 2007 (Chen et al., 2007). This plan aimed to sequence the less complex diploid genomes, which would have direct implications for the more complicated tetraploid cotton species. The prioritization of the D-genome species *G. raimondii* for complete sequencing was a calculated step toward this goal. By 2012, the release of the draft genome sequence of *G. raimondii* marked a pivotal achievement, setting the stage for subsequent characterization of the larger A-diploid and AD-tetraploid cotton genomes, thereby bridging the gap between initial sequencing efforts and the broader application to cotton genomics (Paterson et al., 2012; Wang et al., 2012). This continuum of research and development underscores the interconnected nature of genomic studies, where each phase builds upon the previous, culminating in a comprehensive understanding that benefits functional genomics research.

Diploid species, such as the D-genome (*G. raimondii*) and A-genome (*G. arboreum* or *G. herbaceum*), are considered likely ancestors of the prominent cotton fiber-producing species, *G. hirsutum* and *G. barbadense* (Wendel, 1989). Consequently, *G. raimondii* took the lead as the first species to undergo whole-genome sequencing, with an estimated genome size of approximately 740 Mb (Paterson et al., 2012; Wang et al., 2012). In comparison, the genome assembly of *G. arboreum* is more than twice the size of *G. raimondii* (Li et al., 2014). The draft genome of diploid *G. arboreum* was completed in 2014, employing next-generation sequencing technologies (NGS, Illumina) (Huang et al., 2021).

The successful sequencing and assembly of diploid cotton genomes laid the foundation for unraveling the complexities of allotetraploid cotton species. In 2015, draft genomes of *Gossypium hirsutum* TM-1 were published, utilizing various sequencing platforms such as Illumina and PacBio RSII, along with assembly strategies like 10× genomics+BioNano+Hi-C and PacBio+Hi-C (Li et al., 2015; Zhang et al., 2015). Concurrently, the genome of *Gossypium barbadense* was also sequenced using similar methods (Liu et al., 2015; Yuan et al., 2015).

Recent studies have further refined the assembly of cultivated allotetraploid cotton genomes, including *G. hirsutum* and *G. barbadense*, resulting in high-quality assemblies with improved centromeric regions (Wang M, et al., 2019; Hu et al., 2019). These advancements have paved the way for understanding genomic differences between these two tetraploid cotton species.

Additionally, the genome of *Gossypium turneri* was sequenced using PacBio long reads, Hi-C, and Bionano optical mapping technologies. This effort contributed to the correction of minor assembly errors in the previous *G. raimondii* genome assembly (Udall et al., 2019). The new *de novo* genome assembly of *G. raimondii* and its close relative *G. turneri* was achieved at the chromosome level, enhancing accuracy and correctness compared to the earlier Sanger sequencing-based assembly.

These breakthroughs in cotton genome sequencing hold promise for improving the accuracy and translation of genomics in cotton breeding and genetics.

The recent successful sequencing of multiple cotton genomes has greatly expedited research in cotton functional genomics and population genetics. This achievement has enabled the identification of crucial agronomic trait genes through genome-wide association studies and map-based cloning techniques, thus advancing our understanding of cotton genetics and enhancing breeding efforts. However, mapping data from diverse materials to a single reference genome may lead to the loss of important variants, including presence/absence variation (PAV) and copy number variation (CNV) (Golicz et al., 2016). Consequently, the construction of a population pan-genome through the direct analysis of individual genome sequences presents notable advantages.

The core genome (Core-genome), encompassing genome sequences present in all individuals, is compared with the variable genome (Variable-genome), which includes sequences found in only some individuals (Liu et al., 2020). The crop pan-genome plays a pivotal role in uncovering genetic variations lost during domestication and improvement. This allows breeders to harness core genome variation and rare variation in each material, thereby providing genetic resources to enhance traits such as yield, quality, resistance, and adaptability (Abbas et al., 2022). In 2021, the inaugural cotton pan-genome of allotetraploid cotton was released, featuring the most diverse variation data-set to date, followed by the pangenome of diploid cotton species (Wang et al., 2022; He et al., 2024). It sheds light on the genomics foundations of cotton domestication and improvement across multiple scales, offering valuable insights into essential cotton traits from a pan-genome perspective (Li et al., 2021).

Moreover, Wang et al. (2018) revealed alternative splicing (AS) in polyploid cotton through Pacific Biosciences single-molecule long-read isoform sequencing (Iso-Seq), offering new insight into the complexities and regulation of AS. The swift evolution of sequencing technology, marked by a myriad of sequencing methods and increased precision, robustly bolsters cotton functional genomics research. The creation of mutant populations for broader scientific utility in research and breeding pursuits stands out promising and effective strategy.

In general, prior to large-scale sequencing of genomes, the identification of mutants was limited from a single trait to a single functional gene. However, after the vigorous development of sequencing, large-scale genome-wide mutation identification appeared, which greatly enriched the mutant library and mutant materials, and also greatly promoted the development of forward genetics.

## 3 Genome sequencing revolutionizes plant mutagenesis and crop improvement

Genome sequencing has transformed plant breeding by providing powerful tools like MutMap, MutMap-Gap, Mut-Ren-Seq, and whole-genome sequencing to identify causal mutations underlying desirable traits (Takagi et al., 2015; Chaudhary et al., 2019). Mapping-by-sequencing methods, combining bulked-segregant analysis with NGS data alignment to a reference genome, allow faster gene cloning and candidate gene identification, eliminating the need for extensive marker saturation and physical mapping (Mascher et al., 2014). High-quality reference genomes are crucial for these approaches, providing the genomic context to infer mutation order and position.

Genome sequencing has also enabled precise genome editing tools like CRISPR/Cas9 for targeted mutagenesis in crop plants, offering a more directed approach than random chemical or physical mutagenesis (Nerkar et al., 2022). Mutation rate and pattern analysis from sequencing data has revealed insights like lower mutation frequencies in genes under strong selective pressure, informing effective mutation breeding strategies (Chaudhary et al., 2019). The integration of genome sequencing with mutagenesis techniques has significantly improved functional genomics research in plants. Kubo et al. (2022) employed whole-genome sequencing (WGS) to analyze an MNU-mutagenized rice mutant library, demonstrating the effectiveness of MNU mutagenesis for in silico screening. Sun et al. (2024) used CRISPR/Cas9 and WGS to create and analyze insect-resistant cotton mutants, elucidating molecular pathways contributing to resistance. He et al. (2023) highlighted the potential of CRISPR-based methods for large-scale gene knockout screening and uncovering gene functions and regulatory networks. Wang et al. (2023) combined EMS mutagenesis with exome capture sequencing to create a cataloged wheat mutant library, identifying novel allelic variations in abiotic stress response genes. Zhao et al. (2020) constructed a genome-wide ihpRNA library in Brassica napus using RCA-mediated technology, enabling efficient gene silencing and phenotype identification in this allopolyploid species. Bi et al. (2023) demonstrated the construction of a transcription factor mutagenesis population in tomato using a pooled CRISPR/Cas9 plasmid library and WGS analysis. These studies collectively showcase how genome sequencing has revolutionized mutagenesis approaches, enabling the creation of extensive mutant collections and the understanding of gene functions and regulatory networks underlying important traits. This has significantly accelerated crop improvement efforts by providing insights into the genetic basis of desirable characteristics.

## 4 Advancing crop breeding through mutagenesis

Genetic transformation in crops, such as cotton, is labor-intensive and time-consuming, with a single transgenic event frequently lasting more than a year. Physicochemical mutagenesis has emerged as a viable alternative, eliminating the need for plant genetic transformation and tissue culture techniques.

Mutagenesis techniques, which mix physical and chemical agents, have proven beneficial in crop breeding. Chemical mutagens, such as alkylating agents (e.g., Ethyl Methanesulfonate, Sodium Azide), offer novel methods for creating genetic variations, allowing breeders to research novel traits while boosting crop adaptability, yield, and quality.

Recent advances in genomics technology have expanded the application of mutational breeding. Site-directed mutagenesis, particularly using the CRISPR/Cas9 system, has shown useful in creating targeted modifications in numerous crops, leading to improvements in agronomic metrics such as yield, quality, and stress tolerance (Chen et al., 2017).

To generate superior cultivars, crop breeders typically use induced mutagenesis, which employs physical mutagens such as radiation and chemical mutagens such as EMS. It considerably improves genetic diversity for desired qualities such as stress tolerance and biofortification. Mutagenized populations have been developed for major agricultural species like rice, maize, wheat, and barley (Jankowicz-Cieslak et al., 2017).

Advances in sequencing technologies have made it easier to identify and exploit beneficial mutations induced by mutagenesis. Cloning and inserting desirable mutant alleles into elite germplasm is becoming more feasible, providing a speedy and cost-effective method for producing genetic diversity and improving agriculturally important traits.

In cotton breeding, physical mutagens such as radiation and chemical mutagens such as EMS have been widely used to create mutations that result in heat-resistant, early-maturing, and high-yielding cotton varieties (Zhao et al., 2022). The CRISPR/Cas9 system has emerged as an effective technique for targeted mutagenesis in cotton, allowing for precise gene editing at high efficiency. Mutagenesis in wild cotton species can result in useful traits that can be introduced into cultivated cotton through genetic study and breeding (Kushanov et al., 2022).

In general, mutagenesis, both random and targeted, has proven to be an effective technique for crop breeders to expand genetic diversity and improve agriculturally necessary traits, addressing the global challenge of ensuring food security in the face of rising population and climate change.

## 5 EMS-induced mutant libraries in cotton

In the realm of cotton research, notable advancements have been achieved in the development of EMS-induced mutant libraries within diverse cotton species, such as *G. arboreum* (Kong et al., 2017), *G. barbadense* (Abid et al., 2020), *G. hirsutum* (Lian et al., 2020; Wei et al., 2022), and *G. herbaceum* (Kumar et al., 2022). It is noteworthy that the primary focus of these events centered on conventional cultivated *G. hirsutum* varieties. The key steps and considerations regarding mutagenesis in cotton are outlined below.

### 5.1 Selecting suitable plant materials

The initial phase entails the meticulous selection of appropriate plant materials for mutagenic treatment, placing a strong emphasis on ensuring a high seed germination rate. Significantly, EMS mutagenesis demonstrates broad applicability across various cotton germplasms.

### 5.2 Optimizing EMS concentration and treatment duration

The subsequent crucial step involves conducting preliminary experiments to ascertain the optimal concentration of EMS and the necessary treatment duration. It’s crucial to note that due to the distinctive chemical properties of EMS, utilizing a concentration that is too low may result in an insufficient mutation rate, whereas excessively high concentrations can induce plant mortality and impede seed germination (Abid et al., 2020).

### 5.3 Determining lethal dose

To strike the right balance, researchers commonly calculate the lethal dose (LD50) based on a 50% survival rate under conditions conducive to viable seed production. However, this calculation can pose challenges when dealing with genotypes that do not yield fertile seeds, a phenomenon observed in some instances (Ke et al., 2019; Abid et al., 2020).

### 5.4 Diverse outcomes based on EMS concentration

It’s important to recognize that EMS mutagenesis produces diverse outcomes upon the concentration of EMS applied. Reported concentrations span from as low as 0.3% to as high as 5%, with the majority of experiments falling within the range of 1% to 3%. Despite endeavors to attain an LD50 of 50%, the inherent toxicity of EMS and developmental abnormalities in certain mutant strains often lead to a final M1 generation acquisition rate below 50% (Kong et al., 2017; Lian et al., 2020).

### 5.5 Variability in cotton material sensitivity to EMS

Furthermore, the sensitivity of different cotton materials to EMS significantly influences the frequency of EMS mutagenesis. In terms of treatment concentration, certain studies have employed concentrations as low as 0.3%, while others have reached up to 5%. Nonetheless, most experiments utilize concentrations ranging from 1% to 3%. Irrespective of LD50 calculations, it’s noteworthy that the final M1 generation acquisition rate typically falls below 50% due to EMS toxicity and the occurrence of developmental anomalies (Kong et al., 2017; Lian et al., 2020).

### 5.6 A Plethora of mutant phenotypes

EMS mutagenesis induces a diverse range of mutant phenotypes, influencing various aspects of cotton plants. These variations encompass plant type, leaf and flower shapes, anthers, flower buds, cotton boll characteristics, fiber quality, length, color, as well as resistance to adverse conditions and diseases (refer to **Supplementary Table S1**).

### 5.7 Stabilizing mutant traits

**Figure 1:**
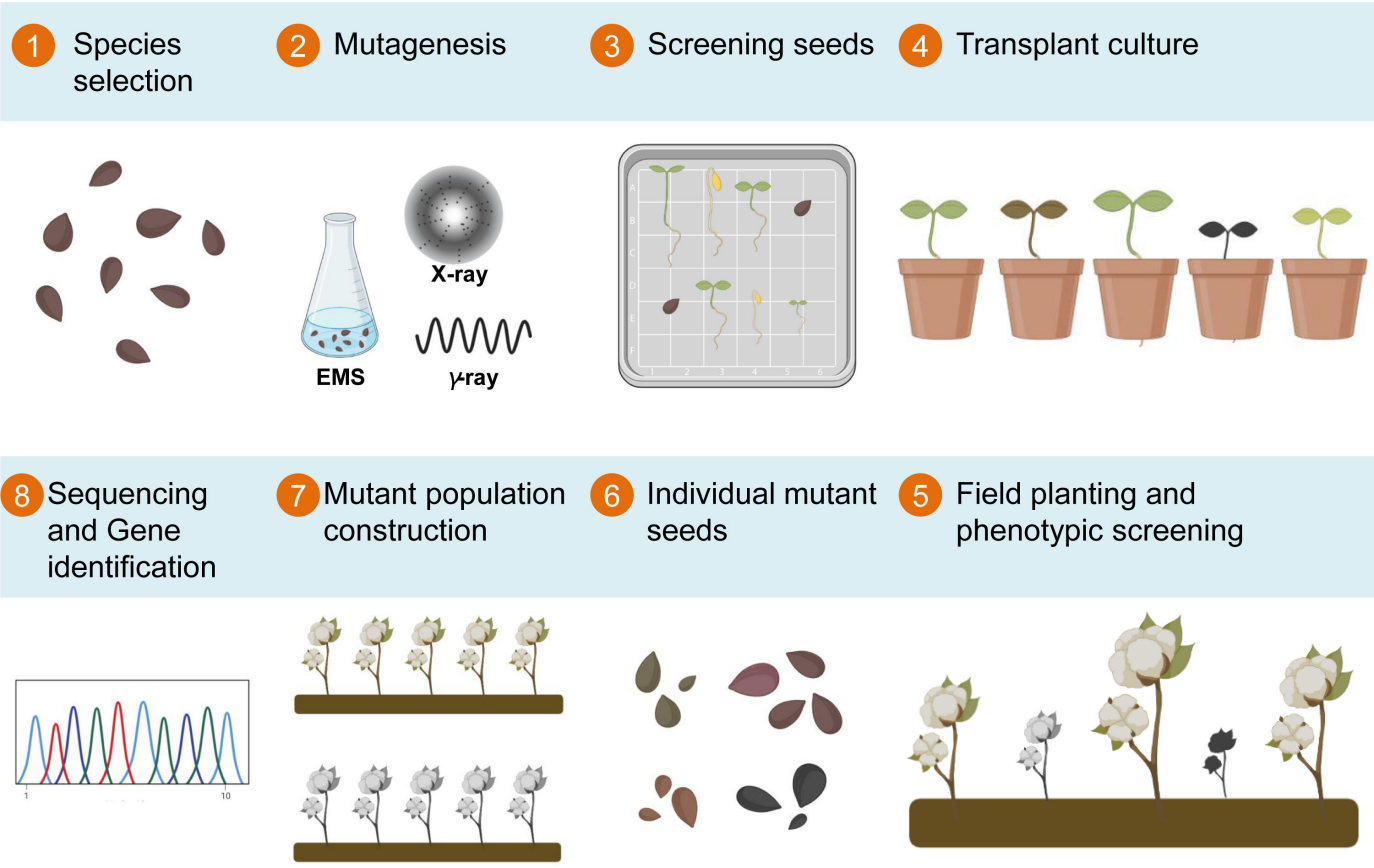
Flowchart illustrating the process of establishing EMS-induced mutant libraries in cotton. The process starts with seed selection (Step 1), followed by the application of a suitable mutagenic treatment, such as EMS, X-ray, or γ-ray (Step 2). Mutated seed screening eliminates damaged and inviable seeds (Step 3), and germinated seeds are transplanted into small pots (Step 4). The selected seedlings are then transplanted to the field for phenotype observation (Step 5), while seeds from plants exhibiting desired traits are harvested (Step 6), forming the mutant population. This mutant population serves as the foundation for mutant sequencing and subsequent gene mapping (Steps 7 and 8, respectively), facilitating comprehensive genetic analysis and gene discovery.

Following the mutagenic treatment, sown seeds frequently yield numerous abnormal plants in the M1 generation. Consequently, multiple generations of breeding become imperative to stabilize mutant traits. Subsequently, the agronomic traits of the mutagenized population are meticulously examined at various growth stages, including cotyledon, seedling, bud, flowering, boll, and spitting stages, to pinpoint variants with distinct phenotypes (**Figure 1**).

## 6 Radiation-induced mutagenesis in cotton breeding

Radiation mutagenesis has played a pivotal role in cotton breeding by inducing favorable traits such as heat resistance and early maturation. Gamma-ray irradiation of cotton seeds has been widely employed for this purpose (Maluszynski et al., 1995). A notable example is the development of the ‘*Lumian1*’ cotton variety, which is distinguished for its high and consistent yield. This variety was derived from hybrid progenies resulting from X-ray radiation mutagenesis applied to ‘*Zhongmian 2*’ and ‘*1195*’ lines (Morgan et al., 1996).

Similarly, to establish a mutant library centered on the cotton inbred line *G. hirsutum* L. TM-1 (Texas Marker-1), linear electron acceleration-based radiation mutagenesis has been employed. Extensive studies have confirmed the stability of TM-1 as a cotton inbred line (Zhao et al., 2022). Interestingly, a distinct investigation on upland cotton pollen grains exposed to 60Co γ-ray gamma-ray revealed noteworthy alterations in their internal structure. While the surface of the pollen remained unaffected, the interior structure underwent remarkable changes, including thinning and irregularities in the interior wall, depolymerization of the endoplasmic reticulum, increased pollen grain inclusions, and a reduced number of pollen tubes in the style. Consequently, these changes resulted in decreased germination rates in the M1 progeny (Yue and Zou, 2012).

Furthermore, the mutant line *‘Zhonghuzhi PI 935’* (referred to as “*PI 935*”) was derived from *G. hirsutum* cv. *Liaomian No. 9* through the utilization of 60Co gamma-ray mutagenesis. *PI 935* not only exhibited favorable traits similar to the original cultivar, such as growing period, drought tolerance, lint color, and fiber quality but also demonstrated higher lint output and yield. Remarkably, compared to control cultivars like *Junmian No. 1* or *Xinluzhong No. 5*, *PI 935* displayed a significantly elevated lint output of approximately 47.3%, making it a promising choice for cotton production (He et al., 2001).

These advancements underscore the potential of radiation mutagenesis in cotton breeding, providing opportunities to enhance cotton fiber properties, yield, and various agronomic traits, thereby contributing to sustainable and improved cotton cultivation.

Although both chemical and physical mutagenesis can bring about mutations in the genome, in contrast, chemical mutagenesis, especially EMS mutagenesis, is currently the most commonly used strategy in plants, mainly based on its relatively simple and rapid operation and good balance of LD50 and mutation frequency.

## 7 High-throughput sequencing for mutant gene identification

**Figure 2:**
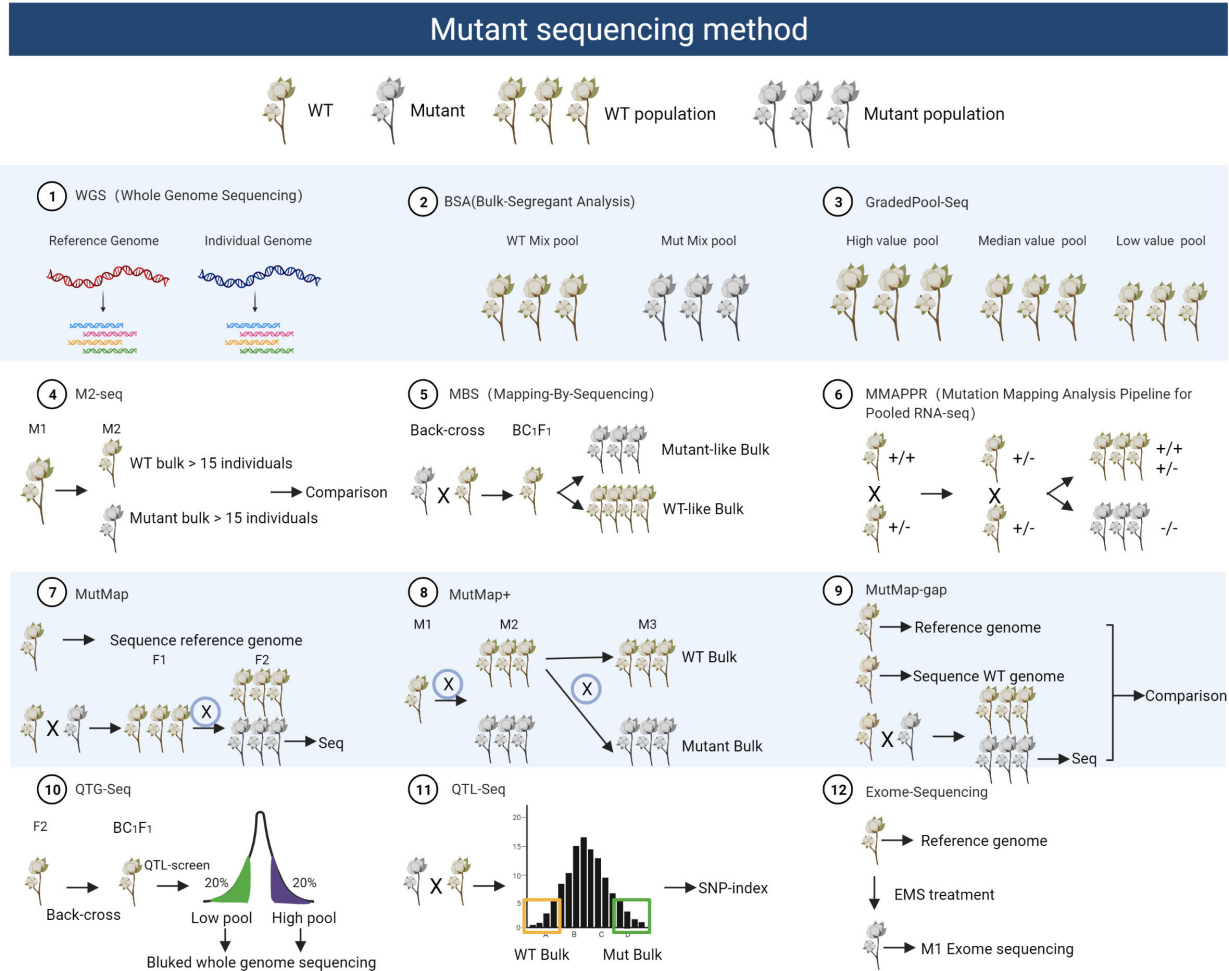
Schematic diagram of different mutant sequencing methods. This figure outlines the steps involved in mutant sequencing methods, from constructing mutant populations to selecting materials and employing various techniques, including Whole Genome Sequencing (WGS), Bulk Segregant Analysis (BSA), Graded Pool-Seq, M2-Seq, Mutant Bulk Segregation (MBS), MMAPPR, MutMap, MutMap+, Mutmap-gap, QTG-Map, QTL-Seq, and Exome Sequencing. Each method serves distinct research purposes, from comprehensive genomic analysis (WGS) to the identification of mutations linked to specific traits (BSA), graded phenotyping (Graded Pool-Seq), or tracking M2 mutations (M2-Seq). These methods offer flexibility in characterizing mutant populations and uncovering genetic variations.

Traditionally, geneticists relied on map-based cloning for the identification of mutant genes, a method involving the labor-intensive process of associating markers with the mutant phenotype and precisely mapping the candidate genes. However, this approach requires large populations and an extensive array of markers, making it time-consuming (Kole and Gupta, 2004). The emergence of high-throughput sequencing has revolutionized mutant gene identification in plants, offering several methods summarized here (**Figure 2**) and their corresponding applications (**Supplementary Table S2**).

One effective approach is Bulked-Segregant Analysis (BSA), which utilizes next-generation sequencing (NGS) to swiftly map genes in plants. BSA facilitates the identification of molecular markers closely linked to causal genes responsible for specific traits (Michelmore et al., 1991). The process involves constructing a segregating population, generating DNA pools from progenies with contrasting phenotypes, and genotyping them with polymorphic molecular markers. These markers guide the further analysis of the linkage between the obtained marker and the target gene position in a known molecular map or chromosome. Cotton research has successfully employed BSA to map important genes (Zhu et al., 2017; Wei et al., 2022).

Building upon BSA, the MutMap method replaces conventional markers with SNPs and utilizes resequencing to directly analyze SNP polymorphisms. This approach involves crossing mutant individuals with their wild-type counterparts to create an F2 population. From this population, mutant phenotypes are selected, and only the DNA from the hybrid pool undergoes high-throughput sequencing. The concept of SNP-index, a commonly used BSA positioning method, is integral to MutMap. It involves fitting SNP-index values to identify genomics intervals associated with the trait, as demonstrated in locating the rice leaf color mutant gene (Abe et al., 2012).

In contrast, MMAPPR (Mutation Mapping Analysis Pipeline for Pooled RNA-seq) deviates from MutMap by sequencing the RNA of extreme individuals within the mutant pool rather than resequencing DNA. This method proves advantageous for larger genomes. It introduces the Euclidean distance (ED) algorithm for trait association analysis within hybrid progeny populations lacking parents. Background noise reduction is achieved through ED power value fitting using LOESS (locally estimated scatterplot smoothing), as exemplified in the analysis of zebrafish cardiovascular mutants (Hill et al., 2013).

MutMap+, an extension of MutMap, ingeniously selects heterozygous individuals at mutation sites for selfing when unexplored mutant individuals cannot develop normally and die at the seedling stage. By sequencing both extreme pools, this method effectively reduces background noise. It also introduces the △SNP-index algorithm for determining trait correlation areas, as demonstrated in locating the rice light green leaf color mutation and validating the function of the OsNAP6 gene’s function (Fekih et al., 2013).

The Mutmap-Gap method combines MutMap and *de novo* assembly to address gene mutations at missing reference gene sites. It involves comparing the wild-type and reference genome to obtain the studied genotype reference genome. Then, using EMS mutagenesis, MutMap analysis identifies the SNP-index map’s peak region genes. If no related genes are found, it implies the mutation site is in the strain-specific gene region. Unmapped reads are *de novo* assembled to identify potential new genes. This method successfully isolated the blast-resistant gene Pii in rice (Takagi et al., 2013a).

QTL-seq complements BSA mapping by focusing on quantitative traits in plants. Specifically, this method selects the reference sequence from the parent with the trait of interest and calculates the SNP-index for trait association using a confidence interval algorithm. The rice blast fungus resistance was mapped using this method (Takagi et al., 2013b). Similarly, QTG-Seq combines genetic map QTL mapping, backcrossing progeny background selection, and QTL-seq to fine-tune the positioning of genes regulating quantitative or qualitative traits and determine the number of main effect sites. It utilizes the F2 genetic map for preliminary mapping, followed by the selection of specific BC1F1 progeny for further analysis and fine positioning. This approach was exemplified in corn plant height, mapping it to the candidate gene Zm00001d020874 (Zhang et al., 2019). Moreover, GradedPool-Seq is an improvement over traditional QTL-seq. Instead of using only two extreme pools, it introduces intermediate phenotype individuals to create “high-value,” “median-value,” and “low-value” groups. Using the Ridit algorithm to calculate allele frequency, this method significantly enhances positioning accuracy. GradedPool-Seq was employed to locate the heterosis QTL GW3p6 (Wang et al., 2019).

Mapping-By-Sequencing (MBS) has accelerated the identification of pathogenic mutations in tomatoes. MBS, reliant on whole-genome sequencing, is a rapid method that takes only 6–12 months from mutant isolation to pathogenic mutation identification. The process involves screening EMS mutants to isolate plants with pathogenic phenotypes, generating an F2 population, and identifying mutated genes through whole-genome sequencing. Utilizing MBS, a yellow-colored tomato fruit mutant was analyzed, revealing a point mutation in the carotenoid pathway gene *PSY1* (Garcia et al., 2016).

In maize and wheat, Exome-Sequencing combines EMS mutagenesis, exon capture, and next-generation sequencing to create mutant libraries. This approach has led to the identification of numerous mutation sites causing amino acid changes, contributing significantly to our understanding of the genetics of these plants’ (Krasileva et al., 2017; Lu et al., 2018).

M2-seq provides a rapid and cost-effective tool for identifying candidate causal mutation sites. It eliminates background mutations through the comparison of M2 populations, and the ΔSNP-index method facilitates effective identification of causal mutations. This approach successfully pinpointed the candidate gene *Glyma.08G193200* in soybean M2 mutant populations (Zhou et al., 2021).

Various sequencing methods for mutant analysis have been demonstrated, with extensive application and validation in model plants like Arabidopsis and rice. Researchers can select the most suitable method based on their specific research objectives and the traits under investigation. For instance, QTL-seq is well-suited for qualitative and quantitative traits influenced by major genes, MutMap is valuable for the analysis of mutagenized mutants, MutMap+ is particularly useful for early lethal mutations or strains that cannot be outcrossed, and MutMap-Gap is designed for scenarios where the target gene is not present in the reference genome (**Figure 2**, **Supplementary Table S2**).

Some of these sequencing strategies are original protocols, and some are based on the original methods to improve and expand to meet the needs of different mutant libraries, and in general, these diverse sequencing techniques have significantly accelerated mutant gene identification in plants. They not only enhance the efficiency of gene mapping but also facilitate a deeper understanding of the genetic basis of various traits, contributing to advancements in plant breeding and genetic research. The choice of method ultimately depends on the specific requirements and nature of the mutant analysis, allowing researchers to employ these tools effectively in their investigations.

## 8 Genes identified by cotton EMS mutant library

Cotton has lags behind model plants like Arabidopsis and rice in terms of whole-genome sequencing, primarily due to its complex polyploid genome. However, recent advancements in functional genomics and mutagenesis research have facilitated the identification of numerous genes and candidate genes associated with key traits in cotton, such as leaf color, plant type, and fiber development (**Supplementary Table S3**).

In relation to leaf color-related genes, researchers conducted crosses on the *Sumian 22* mutant population, observing a 3:1 separation ratio of medium green and green plants in the F2 generation. A specific 0.34 Mb hypermutation interval was identified on the mutant D10 chromosome, encompassing 31 genes. Remarkably, among these genes, only ABCI1 exhibited significantly lower expression levels in mutants compared to the wild type (WT). Simultaneously, the levels of Mg-protoporphyrin IX, prochlorophyll lactone, chlorophyll a, and b in the mutant were markedly reduced in line with the downregulation of *ABCI1*. Furthermore, a critical A to T mutation was identified at -317 bp from the start codon of *ABCI1* in the mutant genome sequence. This mutation likely inhibits *ABCI1* transcription, leading to the green mutation in *Sumian 22*. The reduced transport of protoporphyrin IX to plastids ultimately hinders the synthesis of Mg-protoporphyrin IX, protochlorophyll lactone, and chlorophyll, explaining the observed green phenotype (Gao et al., 2021).

Another leaf color-related gene, *GhCHLI*, was identified through bulked segregant analysis-next-generation sequencing and virus-induced gene silencing strategies. A single nucleotide conversion at position 1366bp (G to A) resulted in the substitution of lysine (K) with arginine (R) at the 361st amino acid, causing the observed change in leaf color (Zhu et al., 2017). Additionally, mapping was used to locate the virescent gene v1, *GhChlI*, which exhibited a non-synonymous nucleotide mutation (G1082A) in its 1269bp coding region. This mutation replaced arginine (R) with lysine (K) in the third exon of Gh_D10G0283. Interestingly, both mutations involve a single amino acid change and impact essential amino acids crucial for optimal growth, nitrogen balance, and fundamental metabolic processes, including photosynthesis, chloroplast biogenesis, and maintenance mechanisms (Alban et al., 2014).

Similarly, for plant type-related genes, researchers successfully cloned the *G. barbadense* axillary flowering (*GbAF*) mutant gene and the upland cotton cluster branch (cl1) mutant gene. Notably, the substitution of aspartic acid (Asp) at position 73 with asparagine (Asn) in the deduced amino acid sequence of *GbAF* led to cotton bolls growing directly on the main plant stem. Dynamic variations in *GhSFT* and *GhSP* levels played a pivotal role in regulating meristems between monopodial and sympodial programs within a single plant (McGarry et al., 2016). These findings suggest that cotton orthologs of *SFT* and *SP* genes can be harnessed to enhance cotton plant architecture (Si et al., 2018).

In the realm of fiber-related genes, a short fiber phenotype mutant was identified within the EMS mutant library, linked to a tetrapeptide repeat-like superfamily protein encoded by Ghir_A12G008870. Gene silencing of Ghir_A12G008870 significantly reduced the fiber length in the WT cotton line MD15 (Fang et al., 2020). Furthermore, researchers discovered a recessive tufted-fuzzless seed mutant on chromosome D04, with a genome interval of approximately 411 kb. Seven genes in this region showed significant differential expression between the tufted lint-free mutant and the wild type. Ghir_D04G019490 emerged as the prime candidate gene due to its proximity to the SNP marker D04_549, which displayed the highest LOD score association with the fuzzless phenotype. Although the exact function of Ghir_D04G019490 remains unknown, this study suggests its involvement in down hair fiber development (Naoumkina et al., 2021). Additionally, researchers characterized a chemically-induced short fiber mutant cotton line, Ligon-lintless-y (liy), controlled by a single recessive locus that affected multiple traits, including plant height and fiber length and maturity. Three candidate genes (2700, 477, and 3260) were identified, showing significant up-regulation in liy at different stages. Gene set enrichment analysis unveiled substantial alterations in various metabolic pathways, such as carbohydrate, cell wall, hormone metabolism, and transport, during liy fiber development (Naoumkina et al., 2017). Additionally, fiber gene expression analysis of 20 selected miRNAs revealed differential expression profiles in short fiber mutants compared to the WT during fiber development, reflecting distinct transcript regulation in mutant lines compared to WT fiber cells. Four miRNA families exhibited significant correlations with fiber length across 11 diverse upland cotton lines (Naoumkina et al., 2016).

The discovery of Ligon lintless-1 (Li1) mutant revealed significantly differentially expressed transcription factors AS2, YABBY5, and KANDI-like in mutant tissues compared to WT tissues. Notably, several down-regulated genes in the mutant leaf transcriptome were related to fiber development, encompassing heat shock protein families, cytoskeleton arrangement, cell wall synthesis, energy metabolism, H_2_O_2_ metabolism-related genes, and WRKY transcription factors (Ding et al., 2014). Moreover, in the mutant GhACT17DM from Li1 plants, the substitution of Gly65 with valine on the nucleotide-binding domain of GhACT17D influenced F-actin polymerization. Compared to the wild-type control, actin filaments in Li1 fibers exhibited higher growth and shrinkage rates, reduced filament skewness, increased filament density, and parallel arrangements (Cao et al., 2021). Similarly, Mapping-by-sequencing unveiled a 22-bp deletion in a pentatricopeptide repeat (PPR) gene that was entirely linked to the immature fiber phenotype in a large F2 plant population and absent in all 163 cultivated varieties tested (Thyssen et al., 2016).

Although the physicochemical mutagenesis technology is relatively simple and it is relatively easy to construct mutant populations, the mutagenesis process is difficult to control, and often a mutant contains more point mutations, which may be caused by the joint action of multiple point mutations, and the mutation phenotype appears. Moreover, after physicochemical mutagenesis, the plant genome may also undergo the rearrangement or deletion of large DNA fragments, and may also promote the transposition of the reverse poson, which will make it more difficult to identify functional genes. At present, most of the functional genes identified by mutants are clearly regulated and observable mutations(leaf color, plant type, fiber, etc.), which also misses a large part of the mutant genes.

## 9 Future directions

### 9.1 Embracing the future: the promise of T-DNA and CRISPR mutant libraries

The insertion mutant library is mainly a mutant library constructed by insertion mutagenesis, and the inserted elements are mainly transposon or T-DNA, and the transposon insertion mutant library and T-DNA insertion mutant library can be created accordingly. Generally, the efficiency of insertion mutagenesis is high, and it has been widely used in the construction of mutant libraries, which has played an important role in functional genomics research. When *Agrobacterium* is used for transformation, through the Ti plasmid, the T-DNA carrying foreign genes infects the plant, through a complex biochemical process, crosses the nuclear membrane, enters the nucleus, and is randomly integrated into the nuclear genome. T-DNA integrated into the nuclear genome can be passed on more stably to the next generation. Different locations where T-DNA is inserted into the genome can cause different genetic mutations, resulting in mutants with different phenotypes. At present, the T-DNA transformation systems of *Arabidopsis thalian*a and rice are relatively mature (Gelvin, 2017; Gong et al., 2021), the library of insertion mutants constructed from T-DNA insertions has played a huge role in the corresponding functional genomics research. However, in some other species, such as maize and cotton, an efficient *Agrobacterium* transformation system has not yet been established, and the *Agrobacterium*-mediated T-DNA transformation process is time-consuming and expensive, and there are often chimeras in the mutants, which increases the difficulty of using T-DNA to construct a mutant library in other species.

**Figure 3:**
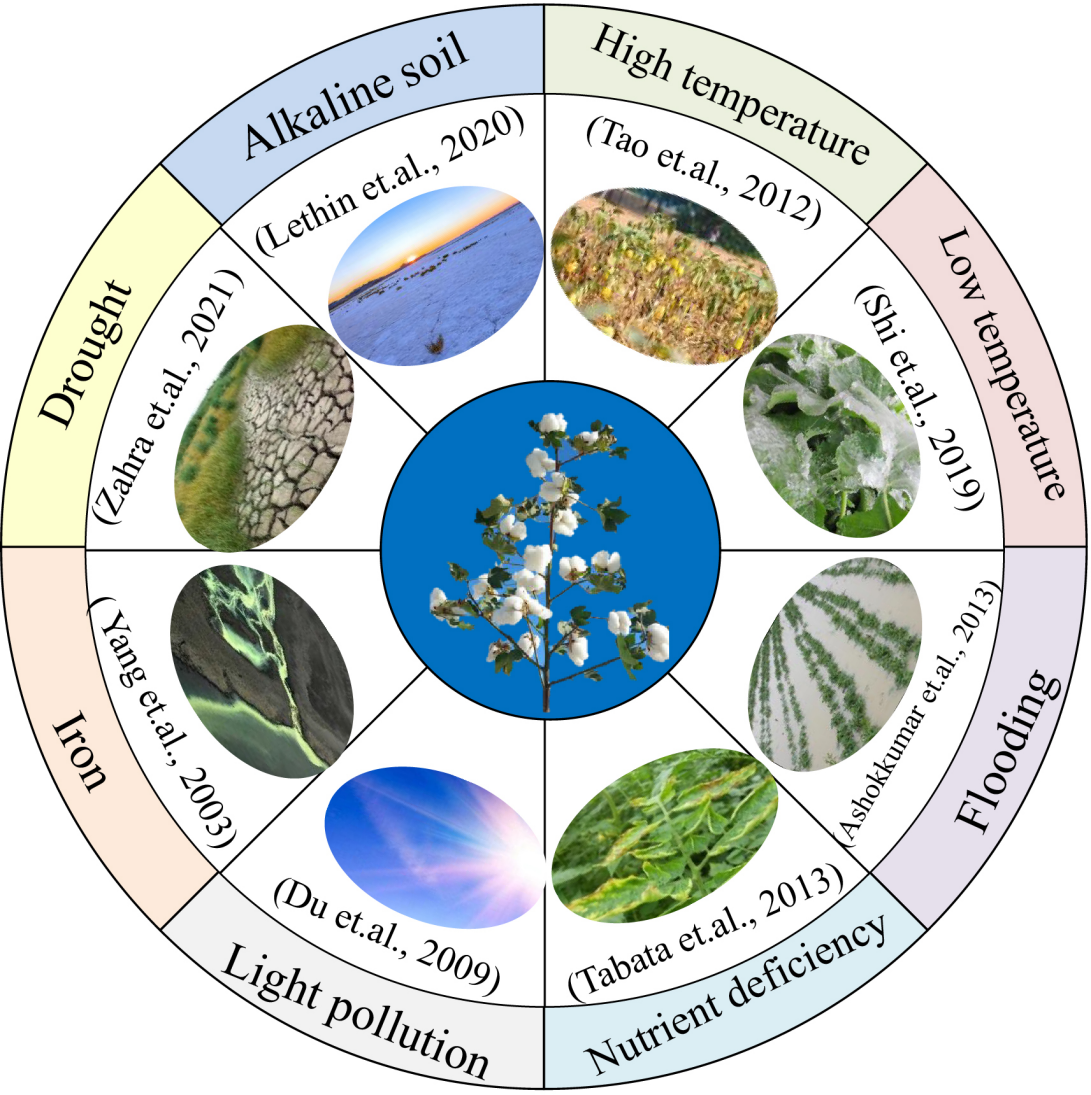
Adaptability of Mutant Plants in Diverse and Challenging Environments This figure highlights the adaptability of mutant plants in diverse and challenging environmental conditions, shedding light on their practical applications to complex scenarios. Mutant materials have proven invaluable in research and applications related to various environmental stressors, including drought, alkaline soil conditions, extreme temperature fluctuations (both high and low), flooding events, nutrient deficiencies, and light pollution. Additionally, these mutants have demonstrated resilience in coping with iron-related challenges. Mutant plants are crucial tools for exploring and enhancing crop adaptability to multiple environmental stresses.

**Figure 4:**
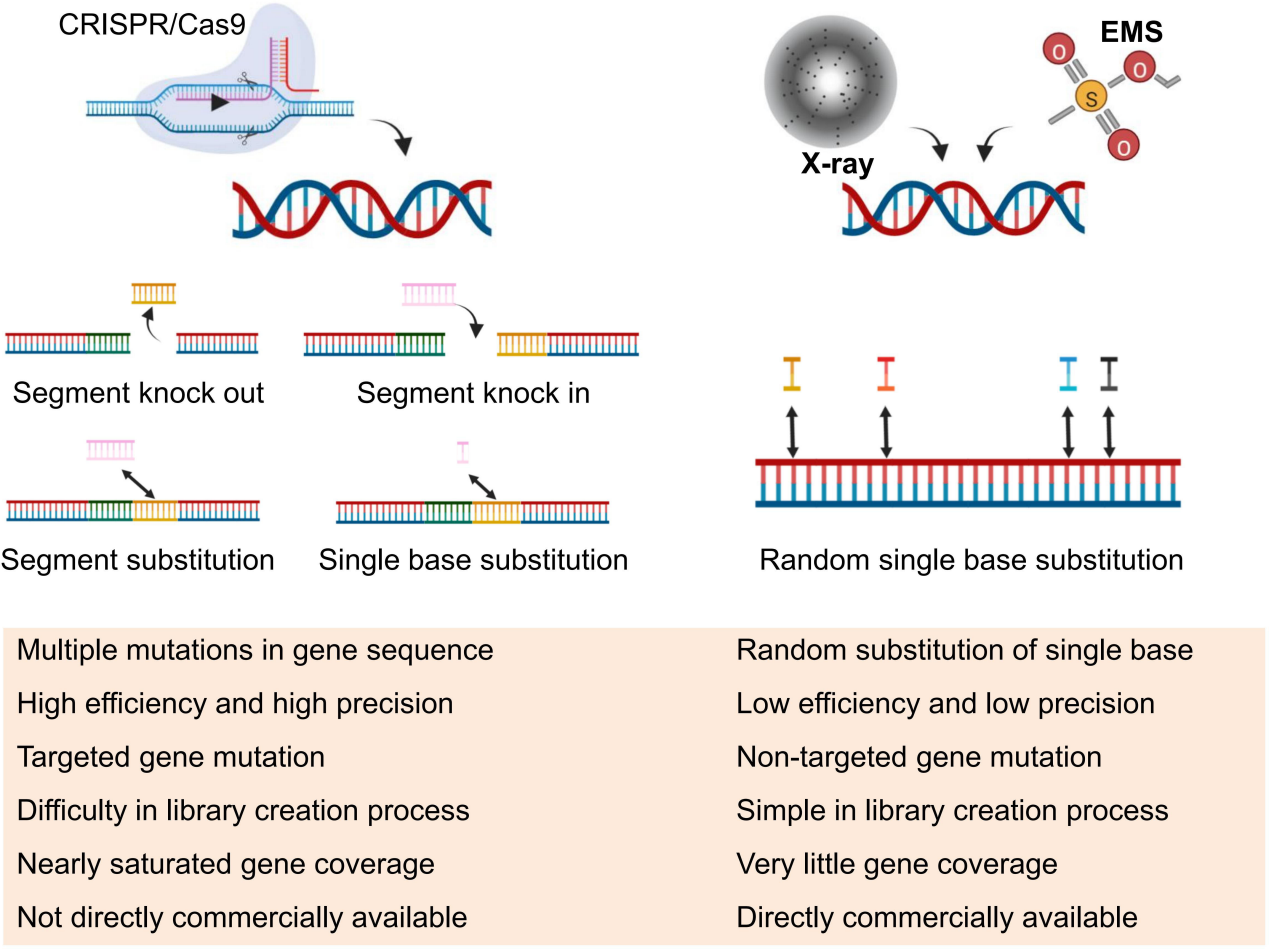
Comparison of CRISPR and Mutagenesis Libraries. In this figure, a concise comparison is presented between CRISPR (Clustered Regularly Interspaced Short Palindromic Repeats) and other mutagenesis libraries, outlining key differences implementation and utilization, offering insights into the distinct approaches employed in genetic research and manipulation.

The future of cotton research is shifting towards the CRISPR mutant library as a promising avenue (**Figure 3**). Currently, various mutants have been screened for resistance genes against different adversities. In addition to the EMS mutant library, other cotton mutant libraries, including the T-DNA insertion mutant library, transposon mutation library, physical mutagenesis via radiation mutants, and the recent CRISPR mutant library (Jia et al., 2012; Zhang et al., 2007; Wang et al., 2013; Ramadan et al., 2021), have been developed. However, the number of mutant cotton plants obtained remains limited. Among the various libraries, the CRISPR mutant library emerges as the foremost choice for prospective mutant library construction, as illustrated in **Figure 4**. Its unique capability to induce targeted and accurate gene mutations presents a compelling advantage, setting it apart from the EMS library, where more than 90% of mutations originate from intergenic regions. In rice, the researchers (Lu et al., 2017; Meng et al., 2017) constructed a total of 91,004 targeted loss-of-function mutants and 12,802 genes mutant libraries, respectively, and although each vector could knock out more than one target gene, it still carried out a considerable scale of transformation. Almost impossible to finish for cotton. In cotton, researchers used a high-throughput gene editing system to create a library of cotton insect resistance gene mutants, but the genes covered by the library were very limited (Sun et al., 2024). In fact, for cotton research, the bottleneck of CRISPR library creation is not the CRISPR technology itself, but the deficiency of the cotton transformation system, which is difficult to transform and has a long cycle, and the only way at present is to transform 2–3 plasmids together to improve the base of the library. Therefore, for cotton and some plants with difficult transformation, the EMS is still the best strategy to obtain large-scale mutagenic resources.

In the realm of cotton genomics, the shortage of resources for reverse genetics in polyploid materials presents a challenge. Conventional cultivated cotton, a heterotetraploid species with a short evolutionary history, features two highly similar sub-genomes with functional redundancy, hindering the identification and selection of recessive mutation traits (Zhang, 2013). Additionally, cotton transformation is a complex and time-consuming process. Establishing a mutant library encompassing numerous genes is crucial for advancing cotton genomics and enriching cotton germplasm resources. Currently, research on mutant cotton germplasm primarily focuses on identifying phenotypic variations, an approach that often overlooks recessive mutations. Therefore, to drive future progress, employing precise sequencing methods to uncover recessive mutations and construct a near-saturation mutant library for cotton is imperative. This endeavor promises to unlock hidden genetic diversity and expedite progress in cotton genomics research.

### 9.2 Harnessing genetic diversity and innovation

**Figure 5:**
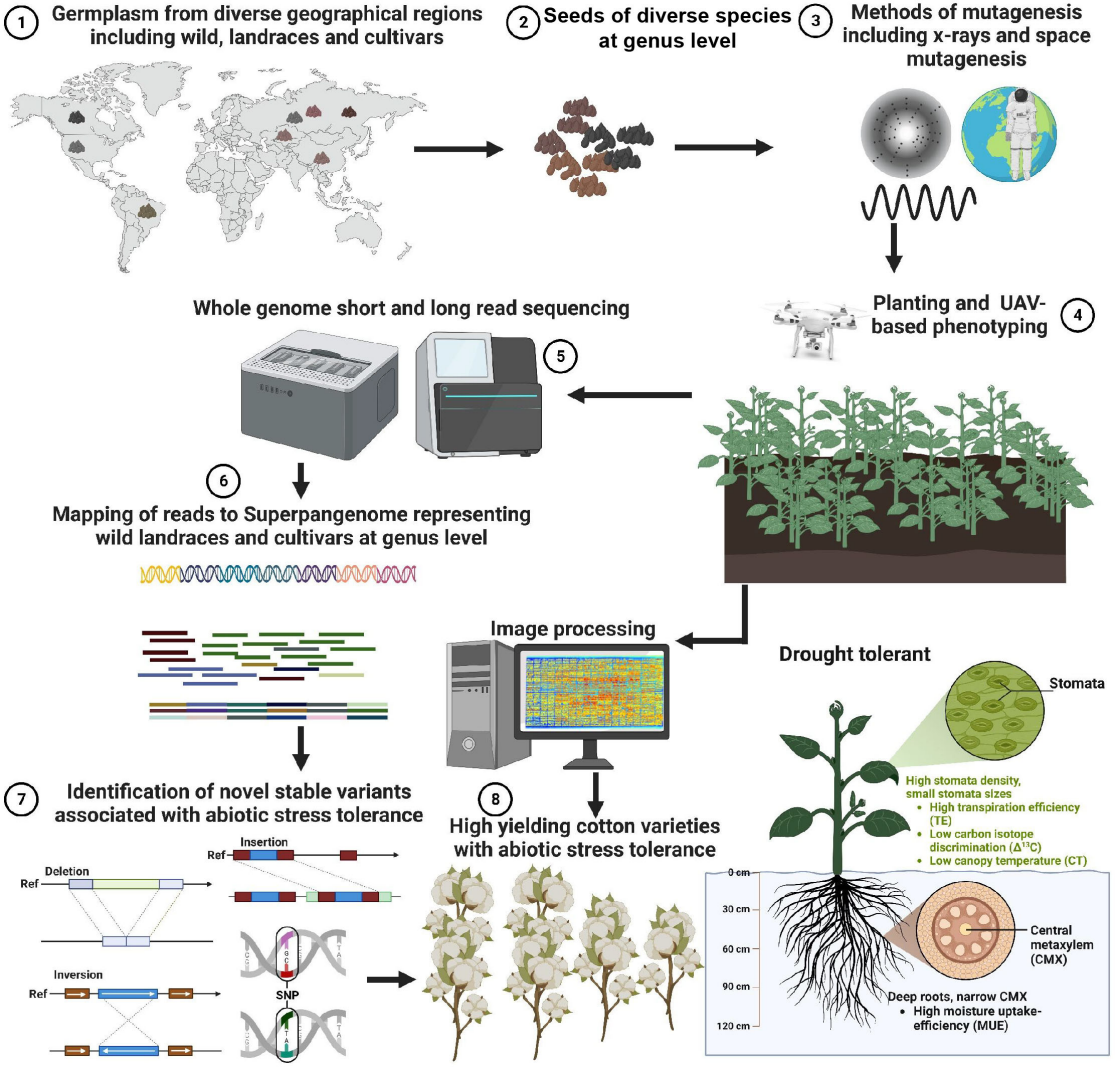
Harnessing Mutation Breeding for Enhanced Stress Tolerance in Cotton Varieties. Mutation breeding offers a promising strategy for the development of cotton varieties resilient to abiotic stress while maintaining high yield potential. To achieve this, a diverse collection of cotton germplasm encompassing wild type, landrace, and cultivar varieties from different global regions is selected at the genus level. Mutations are induced in this germplasm pool through either space-based or x-ray-based mutagenic sources. Subsequently, unmanned aerial vehicle-based (UAV) phenotyping techniques are employed to screen the mutants for specific desired traits. Plants exhibiting the desired phenotypes undergo comprehensive whole-genome sequencing using both long-read and short-read technologies. Notably, emerging super pangenome strategies (Shang et al., 2022) can be employed for read mapping, enabling comprehensive analysis instead of relying on a single linear reference genome. This innovative approach facilitates the identification of novel insertions, deletions, inversions, and single nucleotide polymorphisms (SNPs) associated with stress tolerance, particularly in the context of drought stress induced by climate change.

Mutation breeding is an innovative agricultural approach that holds significant promise for the development of stress-resilient cotton varieties. The core elements of this promising future include the integration of diverse germplasm, the utilization of advanced mutagenic sources, the application of cutting-edge phenotyping techniques via unmanned aerial vehicles (UAVs) (Ye et al., 2023), and the adoption of super pangenome strategies for comprehensive genomic analysis (**Figure 5**). Using a super-pangenome constructed by using different species of cotton at the genus level, representing historical cotton germplasm resources, can also increase the chances of detecting meaningful genetic variants induced by artificial selection.

One critical aspect for advancing mutation breeding lies in the careful selection of a wide-ranging and diverse array of germplasm. This includes wild types, landraces, and cultivars sourced from various regions across the globe. By combining such diversity with the deliberate introduction of mutations, crop adaptability to fluctuating environmental conditions can be significantly enhanced.

Advanced mutagenic sources, such as space-based and x-ray-based techniques, are poised to play a pivotal role in shaping the future of mutation breeding. These sources efficiently induce mutations, allowing for the creation of novel genetic variations that can confer stress tolerance in cotton.

The future of mutation breeding also entails the use of cutting-edge technology for efficient phenotyping. Unmanned aerial vehicles equipped with state-of-the-art phenotyping techniques are set to transform the screening of mutant populations on a large scale. This innovation will enable researchers to swiftly identify and select plants exhibiting the desired phenotypes, thereby expediting the breeding process.

Similarly, comprehensive whole-genome sequencing is vital for unraveling the genetic underpinnings of mutant plants. The adoption of super pangenome strategies, as demonstrated in recent research, opens new horizons for genus-level genetic analysis. This approach not only captures genetic diversity but also reveals genomic complexity, aiding in the identification of novel genetic variants associated with stress tolerance.

Illustrative case studies in rice (Shang et al., 2022) and barley (Jayakodi et al., 2020) underscore the potential of super pangenome strategies. These studies highlight the benefits of pangenomes in capturing genetic diversity and identifying genetic variation, in particular, where frequent inversions were identified from germplasms.

In conclusion, the future of mutation breeding in cotton has the potential to significantly enhance its resilience to environmental challenges while sustaining high productivity levels. This can be achieved through the strategic utilization of diverse germplasms, advanced mutagenic sources, UAV-based phenotyping, the adoption of pangenome strategies, and the application of novel algorithms and machine learning techniques to accurately detect induced mutations, distinguishing them from spontaneous mutations, and thereby uncovering their functional impact on plant phenotypes.

## Author contributions

PW: Conceptualization, Investigation, Writing – original draft, Writing – review & editing. MA: Investigation, Writing – review & editing. JH: Investigation, Writing – review & editing. LZ: Investigation, Writing – review & editing. HC: Investigation, Writing – review & editing. HG: Funding acquisition, Supervision, Writing – review & editing.

## Supplementary material

Supplementary Table 1 EMS mutants in cotton.

Supplementary Table 2 Plant mutant sequencing methods.

Supplementary Table 3 Functional genes identified by mutants in cotton.
