# Supplementary material for: Advances in genome sequencing and artificially induced mutation provides new avenues for cotton breeding
Source: Front Plant Sci. 2024 Jul 2;15:1400201. doi: 10.3389/fpls.2024.1400201 (PMC11250495; doi:10.3389/fpls.2024.1400201)
Supplement: Supplementary file 1 [file DataSheet_1.doc]

1. Aslam, U., Cheema, H.M., Ahmad, S., Khan, I.A., Malik, W., Khan, A.A. 2016. COTIP: Cotton TILLING Platform, a Resource for Plant Improvement and Reverse Genetic Studies. Front Plant Sci. 7, 1863. <https://doi.org/10.3389/fpls.2016.01863.>
2. Bechere, E., Auld, D.L. 2014. Registration of a Tufted-Naked Seed Upland Cotton Germplasm, 9023n4t. J Plant Regist. 8(1), 63–67. <https://doi.org/10.3198/jpr2013.06.0025crg.>
3. Bechere, E., Meredith, W.R., Boykin, J.C. 2013. Registration of Mutant Population MD 15 M4 Gossypium hirsutum L. with Enhanced Fiber Quality. J Plant Regist. 7(2), 216–219. https://doi.org/10.3198/jpr2012.07.0006crg.
4. Brown, N., Smith, C.W., Auld, D., Hequet, E.F. 2013.Improvement of Upland Cotton Fiber Quality through Mutation of TAM 94L-25. Crop Sci. 53(2), 452–459. <https://doi.org/10.2135/cropsci2012.06.0366.>
5. Brown, N., Smith, C.W., Hague, S., Auld, D., Hequet, E., Joy, K., et al. 2015. Within-Boll Yield Characteristics and Their Correlation with Fiber Quality Parameters following Mutagenesis of Upland Cotton, TAM 94L-25. Crop Sci. 55(4): 1513-1523. <https://doi.org/10.2135/cropsci2014.06.0442.>
6. Herring, A.D., Auld, D.L., Ethridge, M.D., Hequet, E.F., Bechere, E., Green, C.J., et al. 2004. Inheritance of fiber quality and lint yield in a chemically mutated population of cotton. Euphytica. 136, 333–339. <https://doi.org/10.1023/B:EUPH.0000032747.97343.54.>
7. Mao, G., Ma, Q., Wei, H., Su, J., Wang, H., Ma, Q., et al. 2018. Fine mapping and candidate gene analysis of the virescent gene v 1 in Upland cotton (Gossypium hirsutum). Mol Genet Genomics. 293(1):249-264. <https://doi.org/10.1007/s00438-017-1383-4.>
8. Mu, G. 2008. Creation of beneficial mutants in cotton and molecular genetic identification of mutant traits (Doctoral dissertation, Hebei Agricultural University). doi: 10.7666/d.y1647920.
9. Muthusamy, A., Jayabalan, N. 2011. In vitro induction of mutation in cotton (Gossypium hirsutum L.) and isolation of mutants with improved yield and fiber characters. Acta Physiol. Plant. 33, 1793–1801. https://doi.org/10.1007/s11738-011-0718-8.
10. Muthusamy, A., Jayabalan, N. 2014. Radiation and chemical mutagen induced somaclonal variations through in vitro organogenesis of cotton (Gossypium hirsutum L.). Int J Radiat Biol. 90(12), 1229–1239. https://doi.org/10.3109/09553002.2014.923589.
11. Muthusamy, A., Vasanth, K., Jayabalan, N. 2005. Induced high yielding mutants in cotton(Gossypium hirsutum L. ). IAEA. 2005(1):6-8. <https://doi.org/10.37992/2021.1204.146>.
12. Patel, J.D., Wright, R.J., Chandnani, R., Goff, V.H., Ingles, J., Paterson, A.H. 2016. EMS-mutated cotton populations suggest overlapping genetic control of trichome and lint fiber variation. Euphytica, 208(3), 597–608. <https://doi.org/10.1007/s10681-015-1614-x.>
13. Ye, C., Zhuang, Z., Xie, Z. 2015. EMS-induced cotton generations identified and validated by molecular markers. 2015, Vol. 13. 1730–1734. doi: 10.13271/j.mpb.013.001730.
